# Assessment of the Economic Structure of Brazilian Agribusiness

**DOI:** 10.1155/2016/7517806

**Published:** 2016-05-03

**Authors:** Vilmar Rodrigues Moreira, Ricardo Kureski, Claudimar Pereira da Veiga

**Affiliations:** ^1^Pontifical Catholic University of Paraná (PUCPR), Rua Imaculada Conceição, 1155 Bloco Acadêmico, Sala 103B, Prado Velho, 80215-901 Curitiba, PR, Brazil; ^2^Federal University of Paraná (UFPR), Avenida Prefeito Lothário Meissner, No. 632, 80210-170 Curitiba, PR, Brazil

## Abstract

This paper presents an economic assessment of Brazilian agribusiness and its relationship with other economic sectors. It was found that, in 2011, agribusiness had a share of 18.45% (basic prices) and 19.77% (market prices) of Brazilian GDP. The tax burden of agribusiness (20.68%) was higher than that of other sectors (13.59%), despite agribusiness being a major contributor to the generation of foreign exchange, employment, and essential products, such as food. Brazilian agribusiness is a major employer, responsible for 29.39% of national employment. However, its average income is lower than in the other sectors of the Brazilian economy. Finally, agribusiness was found to be the biggest generator of foreign exchange, with a positive balance of trade. It was possible to conclude that agribusiness forms a strong link between agriculture and livestock, industry, and services in other economic sectors. For this reason, it can be said that the development of agribusiness is highly relevant to the process of Brazilian economic development and is therefore important to the progress of economic policies.

## 1. Introduction

The concept of agribusiness firstly appeared on the seminal work of Davis and Goldberg in the book* A Concept of Agribusiness* [1, 2]. It is a comprehensive concept and includes, in addition to rural property, all production, support, and agricultural distribution activities [3, 4]. It is also a system of production chains that encompasses suppliers of materials and services, farms responsible for production (crops, livestock, and plant extracts), storage, processing, and manufacturing industries, and distribution and marketing agents [5, 6]. The interaction and influence between the links of the chain are critical to the concept of agribusiness. Adding value to industry's products involves five distinct stages: (i) supply, (ii) production, (iii) processing, (iv) storage, and (v) distribution. According to the Brazilian Corporation of Agricultural Research (Empresa Brasileira de Pesquisa Agropecuária (EMBRAPA) https://www.embrapa.br/), agribusiness is a network composed of several agents that are responsible for the production and sale of inputs, agricultural production, processing, distribution, and sale to the end-consumer [7, 8].

**Figure 1:**
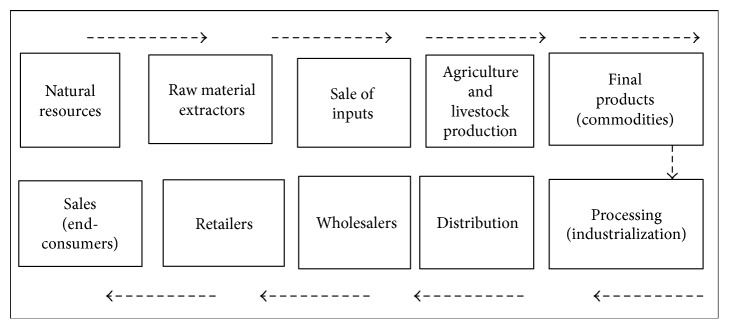
Agribusiness supply chain.

Figure 1 illustrates agribusiness as a comprehensive supply chain.

The production and sale of inputs involve the extraction of raw materials, processing, and distribution, leading to sale for agricultural production. Agricultural production by small, medium, and large producers involves technical support, environmental management, and other direct and indirect aspects that are related to the generation of goods and services in the rural environment. Processing, distribution, and sale encompass industry, distributors, and consumers of agricultural products and services. Agribusiness also includes the institutional environment, which consists of the culture, traditions, education, and customs, in addition to the organizational environment, composed of information, associations, research and development, and finance [7, 9, 10].

Agribusiness has always played a key role in the development of the Brazilian economy [11]. Brazil's economic upturns during the coffee, cattle, sugarcane, sugar, rubber, cocoa, and other “cycles” are proof of this industry's economic and social importance [12]. According to Guilhoto et al. [13], the country's economic tradition in agribusiness is a trend that should prevail in the future, primarily because of the availability of its vast natural resources. The size of Brazilian territory is 880,000,000 hectares, of which 388,000,000 is arable, and 90,000,000 has yet to be explored [14]. This availability of area, nonexistent in most countries, coupled with the global growth in food demand, creates a positive scenario for Brazilian agribusiness. The industry employs 38% of the country's workforce and accounts for approximately 40% of the volume of national exports [15]. Agribusiness is an economic industry of vital importance to Brazil because it is responsible for a significant share of job creation, positively supports the balance of trade with the strength and magnitude of its exports, and substantially influences the composition of Brazilian GDP [11].

Due to the wide-ranging economic influence of agribusiness and its intersector relationships, the performance of this sector has been highlighted as a critical component of the economic development of regions where agribusiness has a considerable share in economic activity [16, 17]. This is the case of Brazil, where agribusiness historically has a large share in the national economy. Between 2006 and 2011, the representation of agribusiness hovered around 23% [18].

In order to assess the economic context of agribusiness activities and their impact on regional development [19], this paper presents the estimation and analysis of the GDP, direct taxes, employment, wages, and balance of trade of Brazilian agribusiness in 2011. These results highlight impact of agribusiness on the economic system and the main elements to be considered in the process of improving economic policies for major regional development.

## 2. Materials and Methods

### 2.1. Data Sources and Methodological Procedures

Agribusiness is a set of activities of supply, goods, and services that are conveyed and sold to end-consumers. Thus, it is not possible to measure directly the GDP, taxes, employment, and wages of agribusiness due to the chaining involved in its many activities. First, it is necessary to identify this chaining using an input-output matrix. In 2011, the official Brazilian statistics department (Instituto Brasileiro de Geografia e Estatística (IBGE)) released the “table of supply and uses” as an integral part of the compilation of national accounts. Using this table and the methodology of Guilhoto and Sesso Filho [20], it was possible to estimate the Brazilian input-output matrix.

The methodology that was used to estimate the GDP, taxes, employment, and wages in agribusiness is based on the works of Finamore and Montoya [21] and Guilhoto et al. [22]. In this section, we present an explanation of the input-output matrix and the methodology for estimating the GDP, taxes, employment, and wages of agribusiness.

### 2.2. Input-Output Matrix

The input-output matrix is a statistical double entry table. It covers the register of supplies for economic activities and the destination of produced goods. This record provides the perception of sectorial interdependence.

According to Polenske [23], transactions are placed like a matrix in the input-output table with each cell representing a sale and a purchase at the same time. In each row, sales from an industry to each intermediary are noted.

Table 1 shows a simplified structure of an input-output matrix. Goods and services that are meant for intermediate and final demand are represented in the rows. In the columns, we can see the total intermediate demand acquired by economic activities for the production of other goods and services. If we deduct the production total from the intermediate consumption, we can arrive at the value added which remunerates the production factors.

In the input-output matrix, the gross value of the production of a sector *i* (*X*_*i*_) is given by

(1) $$\begin{array}{c} \sum x_{ij}+\sum Y_{i}=X_{i}. \end{array}$$

Defining the technical coefficient (*a*_*ij*_) as supplies by unit of the gross value of production of sector *i* and replacing it on expression (1), we have

(2) $$\begin{array}{c} a_{ij}=\frac{x_{ij}}{X_{i}}. \end{array}$$

Using a matrix notation, we can rewrite expression (1) as follows:

(3) $$\begin{array}{c} X=AX+Y, \end{array}$$

where *X* is vector of the gross value of production, *A* is technical coefficient matrix, and *Y* is vector of final demand.

By isolating the gross value of production, it is possible to calculate the direct and indirect effects as the result of increasing a unit in final demand. The result of this operation is the Leontief matrix as follows:

(4) $$\begin{array}{c} X={\left(\;I-A\;\right)}^{-1}Y, \end{array}$$

where (*I* − *A*)^−1^ is called the matrix inverse of Leontief or total impact matrix or even the matrix of direct and indirect needs.

### 2.3. Agribusiness GDP

In order to calculate agribusiness GDP, it is necessary to consider the entire production chain. Therefore, agribusiness is divided into four segments: inputs, agriculture and livestock, industry (agriculture-based), and distribution (transport, trade, and services). In each segment, the GDPs corresponding to the sectors of agriculture (total production chains of crops and other plant activities) and livestock (total production chains of animal products) are estimated separately and then aggregated according to the following classification: Aggregate I: inputs; Aggregate II: agriculture and livestock; Aggregate III: agroindustry; and Aggregate IV: distribution and services. The end-result of the agribusiness GDP is the sum of the four aggregates [18].

To calculate the GDP of Aggregate I, it is necessary to calculate the coefficient of value added. This procedure is mandatory to avoid the multiple count error because the value provided is not just the value added, but also a share of the production value that is provided to the activity by the other sectors. The coefficient of value added is calculated as follows for each input supplier sector:

(5) $$\begin{array}{c} {CVA}_{i}=\frac{{VA}_{i}}{X_{i}}, \end{array}$$

where CVA_*i*_ is coefficient of value added of sector *i*; VA_*i*_ is value added; and *X*_*i*_ is total production.

If we multiply (5) by the total value of input supply, we obtain the GDP for input supply as follows:

(6) $$\begin{array}{c} {GDP}_{I}=\sum Z_{i}*{CVA}_{i}, \end{array}$$

where GDP_I_ is GDP of Aggregate I (inputs) of agriculture, forestry, logging and livestock, and fisheries; *Z*_*i*_ is total value of input supply of the sector *i*; and CVA_*i*_ is coefficient of value added.

Aggregate II corresponds to the agriculture and livestock GDP, which is calculated by multiplying the gross value of production by the value added as follows:

(7) $$\begin{array}{c} {GDP}_{II}=X_{i}*{CVA}_{i}, \end{array}$$

where GDP_II_ is total agriculture and livestock GDP; *X*_*i*_ is total of production of sector *i*; and CVA_*i*_ is coefficient of value added.

To measure the GDP of agriculture and livestock-based industry, which constitutes the GDP of Aggregate III, it is necessary to estimate the value added of each agroindustrial segment. The industrial segments that consume raw materials from agriculture in the input-output matrix were determined. The sectioned sectors were food and beverages, textiles, clothing items and accessories, leather goods and footwear, and wood products—excluding furniture, cellulose and paper products, alcohol, pesticides, furniture, and products of various industries. To avoid double counting, the value of the supply of inputs to agriculture, computed in Aggregate I, was subtracted from the agricultural industry's value added. Thus, Aggregate III may be calculated using the following equation:

(8) $$\begin{array}{c} {GDP}_{III}=\sum \left(\;{VA}_{i}-Z_{i}*{CVA}_{i}\;\right), \end{array}$$

where GDP_III_ is GDP of Aggregate III (agroindustry); VA_*i*_ is value added of sector *i*; *Z*_*i*_ is total value of input supply; and CVA_*i*_ is coefficient of value added.

The GDP of Aggregate IV is the share of agricultural GDP related to distribution and services. To this end, first, the value added of trade, transport, and services was obtained. For services, the value added of the following service activities was considered: information, financial intermediation and insurance, real estate and rent, boarding and lodging, and services provided to businesses. The value added was calculated by adding the value added to net indirect taxes of subsidies on products. It was also necessary to consider the values of the final demand of the agribusiness segment when totaling the final domestic demand. Therefore, the final domestic demand was obtained using the following equation:

(9) $$\begin{array}{c} DFD=GFD-NITFD-IPFD, \end{array}$$

where DFD is domestic final demand; GFD is global final demand; NITFD is net indirect taxes paid on the final demand; and IPFD is imported products on the final demand.

Thus, the GDP of Aggregate IV was calculated as follows:

(10) GDPIV=VAC+VAT+VAS−Z∗CVA∗∑FDiDFD,

where GDP_IV_ is GDP of Aggregate IV (distribution and services); VAC is value added of commerce; VAT is value added of transportation; VAS is value added of services; *Z* is total value of input supply; CVA is coefficient of value added; FD_*i*_ is final demand of agribusiness activities; and DFD is domestic final demand.

The total agribusiness GDP corresponds to the sum of the GDPs of the four aggregates, that is, the sum of the results of (6), (7), (8), and (10). Consider

(11) $$\begin{array}{c} {GDP}_{agribusiness}={GDP}_{I}+{GDP}_{II}+{GDP}_{III}+{GDP}_{IV}. \end{array}$$

### 2.4. Agribusiness Employment

The procedure for estimating the level of agribusiness employment is similar to the procedure for estimating GDP [21]. The first step is to calculate the coefficient of labor as follows:

(12) $$\begin{array}{c} {CL}_{i}=\frac{L_{i}}{X_{i}}, \end{array}$$

where CL_*i*_ is coefficient of labor of sector *i*; *L*_*i*_ is number of workers; and *X*_*i*_ is total production.

The total of employees for supplying inputs for agriculture, forestry, logging, and livestock and fisheries is calculated as follows:

(13) $$\begin{array}{c} E_{I}=\sum Z_{i}*{CL}_{i}, \end{array}$$

where *E*_I_ is number of employees of Aggregate I (inputs); *Z*_*i*_ is total value of input supply of the sector *i*; and CL_*i*_ is coefficient of labor.

Employment for agriculture and livestock is calculated as follows:

(14) $$\begin{array}{c} E_{II}={VBP}_{i}*{CL}_{i}, \end{array}$$

where *E*_II_ is number of employees of Aggregate II (agriculture and livestock); VBP_*i*_ is gross value of production of sector *i*; and CL_*i*_ is coefficient of labor.

For the agriculture and livestock-based industry, the total number of employees of each sector is calculated by discounting the employees of supply of inputs (see (13)). Consider

(15) $$\begin{array}{c} E_{III}=\sum \left(\;E_{i}-Z_{i}*{CL}_{i}\;\right), \end{array}$$

where *E*_III_ is number of employees of Aggregate III (agroindustry); *E*_*i*_ is employees of agroindustry of sector *i*; *Z*_*i*_ is total value of input supply; and CL_*i*_ is coefficient of labor.

The total number of employees for distribution and service is calculated considering the number of employees of trade, transport, services, supply of inputs, and final demand. Consider

(16) $$\begin{array}{c} E_{IV}=\left(\;\text{LC}+\text{LT}+\text{LS}-Z*\text{CL}\;\right)*\sum \left(\;\frac{{FD}_{i}}{\text{DFD}}\;\right), \end{array}$$

where *E*_IV_ is number of employees of Aggregate IV (distribution and services); LC is number of workers of trade sector; LT is number of workers of transportation sector; LS is number of workers of service sector; *Z* is total value of input supply; CL is coefficient of labor; FD_*i*_ is final demand of agribusiness activities of sector *i*; and DFD is domestic final demand as calculated in (9).

Thus, the total number of agribusiness employees is calculated by adding the aggregates. Consider

(17) $$\begin{array}{c} E_{agribusiness}=E_{I}+E_{II}+E_{III}+E_{IV}. \end{array}$$

### 2.5. Agribusiness Wages

Like the procedure to estimate the level of agribusiness employment, the procedure for estimating agribusiness wages is similar to the procedure for estimating GDP. First, it is necessary to calculate the coefficient of wages. Consider

(18) $$\begin{array}{c} {CW}_{i}=\frac{W_{i}}{X_{i}}, \end{array}$$

where CW_*i*_ is coefficient of wages of sector *i*; *W*_*i*_ is wage income; and *X*_*i*_ is total production.

Wages related to the supply of inputs for agriculture, forestry, logging, and livestock and fisheries are calculated by multiplying the total value of input supply by the coefficient of wages. Consider

(19) $$\begin{array}{c} W_{I}=\sum Z_{i}*{CW}_{i}, \end{array}$$

where *W*_I_ is wages of Aggregate I (inputs); *Z*_*i*_ is total value of input supply of the sector *i*; and CW_*i*_ is coefficient of wages.

The wage of agriculture and livestock is calculated as follows:

(20) $$\begin{array}{c} W_{II}={VBP}_{i}*{CW}_{i}, \end{array}$$

where *W*_II_ is wages of Aggregate II (agriculture and livestock); VBP_*i*_ is gross value of production of sector *i*; and CW_*i*_ is coefficient of wages.

For agriculture and livestock-based industry, the wage of each sector is calculated by discounting the wage of supply of inputs (see (19)). Consider

(21) $$\begin{array}{c} W_{III}=\sum \left(\;W_{i}-Z_{i}*{CW}_{i}\;\right), \end{array}$$

where *W*_III_ is wage of Aggregate III (agroindustry); *W*_*i*_ is wages of agroindustry of sector *i*; *Z*_*i*_ is total value of input supply; and CW_*i*_ is coefficient of wages.

The wage for distribution and service is calculated considering the wage of trade, transport, services, and supply of inputs and final demands. Consider

(22) $$\begin{array}{c} W_{IV}=\left(\;\text{WC}+\text{WT}+\text{WS}-Z*\text{CW}\;\right)*\sum \left(\;\frac{{FD}_{i}}{\text{DFD}}\;\right), \end{array}$$

where *W*_IV_ is wage of Aggregate IV (distribution and services); WC is wage of trade sector; WT is wage of transportation sector; WS is wage of service sector; *Z* is total value of input supply; CW is coefficient of wage; FD_*i*_ is final demand of agribusiness activities of sector *i*; and DFD is domestic final demand as calculated in (9).

Thus, the wage of agribusiness is calculated by adding its aggregates. Consider

(23) $$\begin{array}{c} W_{agribusiness}=W_{I}+W_{II}+W_{III}+W_{IV}. \end{array}$$

## 3. Brazilian Agribusiness in the Structure of GDP and Taxes

Considering basic prices, agribusiness represented 18.45% of Brazilian GDP in 2011 (see Table 2). The results show that in agribusiness GDP a share of 27.78% is due to agriculture and livestock (Aggregate II). This implies that rural activity is strongly linked to urban sectors and therefore interconnected with the rest of the economy, since the remaining agribusiness GDP (72.22%) is due to activities outside of rural areas. Therefore, agriculture and livestock can be considered key sectors in Brazil and closely linked to the national economy. Regarding market prices, which consider net indirect taxes on activities, the share of agribusiness in Brazilian GDP grows to 19.77% (1.32% higher). It is also possible to highlight an increase of 2.07% in the share of Aggregate III (agroindustry) in Brazilian GDP, if we compare basic and market prices.

The analysis of indirect taxes (Table 3) allows us to identify where the Brazilian government focuses on tax collection. Considering the total taxes, agroindustry (Aggregate III) and industry (Aggregate V) paid more because their taxes on primary factors of production (share in total) were 18.80% and 52.62%, respectively. Considering absolute values, the agribusiness sector paid less income tax than the rest of the economy because it is less representative in economic terms. However, in relative values, the tax burden of agribusiness (20.68%) was higher than the tax burden of other sectors of the Brazilian economy (13.59%). According to Finamore and Montoya [21], this situation is paradoxical, since agribusiness is a major contributor to the generation of foreign exchange, employment, and essential products, such as food. Still, according to the authors, historically, the relationship between agribusiness and the urban-industrial sectors has followed this pattern of withdrawing economic surpluses from agribusiness to the development of urban-industrial sectors.

**Figure 2:**
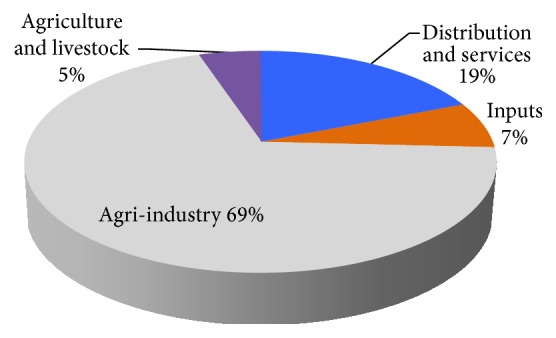
Share of agribusiness aggregates in indirect taxes.

Figure 2 shows the distribution of taxes paid by agribusiness. Agroindustry and services were the most representative, with a share of 88.26% of the total.

## 4. Agribusiness Employment

Historically in Brazil, agribusiness has been a major generator of employment. In 2011, 29.39% of national employment was due to agribusiness (Table 4).

Considering the share of agribusiness in total Brazilian GDP (Table 2), it is possible to highlight that the importance of agribusiness concerning employment is higher than in value added either in basic or in market price-based analysis. It can also be shown that the activities of the other economic sectors are based more on capital than on manpower.

The intense use of manpower in Brazilian agribusiness can be acknowledged if we analyze the share of the aggregates in total agribusiness employment. Agriculture and livestock (Aggregate II) have a share of 49.13%, which is almost the same joint share (48.55%) of agroindustry (Aggregate III) and distribution and services (Aggregate IV).

## 5. Agribusiness in Brazilian Wage Structure

By relating information on workforce with wage income, it can be seen that average income in agribusiness was lower than in the remaining sectors of the Brazilian economy. While the average annual wage income per agribusiness worker was BR R$ 8,027.40 or 14.73 units of national minimum wages (MWs), the average in the other sectors was BR R$ 17,337.22 or 31.81 MWs (Table 5).

Considering the analysis by all aggregates, workers in agriculture and livestock (Aggregate II) have the lowest wage income (4.29 MWs), while workers in industry services (Aggregate VI) have the highest (43.19 MWs). This is true even among aggregates of agribusiness. In other words, this discrepancy between wages may be related to the higher or lower degree of qualification of the workforce.

Although some studies of wage differences show that the agriculture and livestock workforce is usually less qualified than those in the urban sector, there is no consensus concerning this relationship between the industry and of services workforce. Nevertheless, if we consider the hypothesis of the higher the qualification, the higher the remuneration and if we consider the wage of aggregates related to industry (agroindustry and industry) and services (distribution and services, industry services, and services), then we see that these sectors have higher wage income and, consequently, a more qualified workforce.

Another way to assess wage income is by analyzing how much of the value added is appropriated by the workers. In Table 5, we analyze the share of the aggregates in the GDP of aggregates (basic prices). Among the aggregates, the workers in agroindustry and services have the higher share. Workers in industry services and agriculture and livestock have the smallest share of GDP. Concerning agriculture and livestock workers, if we jointly consider data on the average annual wage income, we can infer that the use of the workforce is more intensive than in other sectors

## 6. Brazilian Balance of Trade

One of the aims of analyzing input-output structure is to understand the interdependence relationships with foreign trade. In economic systems, imported goods are used as inputs on intermediate consumption or consumed as products in final demand (even though they are available domestically). Imports represent the expenses that elude regular income, since a share of expenses is not applied with goods produced domestically. Therefore, it is important to analyze the contributions of sectors to balance of trade, considering either imports or exports by source and destination.

Brazilian imports in 2011 are shown in Table 6. In the import structure, 65.4% of the total is intended for intermediate consumption and 34.6% is for final demand. The distribution shows a high level of dependency on imported goods to the production supply. This dependency is higher in the services (74%) sector and more balanced in agribusiness (52.7%).

Table 7 shows the share of sectors in imports and the Brazilian balance of trade. The industry sector is the major importer, with 65.88% of the total. The results of the balance of trade show that the agribusiness sector is the biggest generator of foreign exchange, although not enough to balance the net value in 2011. The data also show that when compared to the industry and service sectors, agribusiness has a competitive advantage. Industry and services had a deficit in the balance of trade, whereas agribusiness had a large surplus. It can also be seen that agribusiness had a better balance in foreign trade than the service sector.

In short, the assessment of the balance of trade shows that the Brazilian economy is relatively closed to foreign trade considering the amount of imports to final demand. It can be inferred that there is room for programs that move toward the replacement of imports in industry, especially considering intermediate consumption. This might be feasible through the development of better national industrial policies. Furthermore, it is important to highlight the importance of agribusiness sector to the balance of trade and, thus, to the Brazilian economy.

## 7. Conclusions

This paper aimed to assess the economic dimension of Brazilian agribusiness in 2011 considering GDP, taxes, employment, wages, and foreign trade. Agribusiness was seen to represent 18.45% of Brazilian GDP (basic prices) and 19.77% (market prices), indicating that a large share of Brazilian economic development is due to agribusiness activities. The analysis of agribusiness aggregates showed that its activities are more integrated with urban sectors, since the largest share of agribusiness GDP is formed by agroindustry and services.

On the assessment of the tax structure, the tax burden of agribusiness was shown to be higher than in other sectors, despite agribusiness being a major contributor to the generation of foreign exchange and employment and essential products, such as food.

Concerning employment, historically agribusiness has been a major generator of employment in Brazil. In 2011, the sector was responsible for 29.39% of national employment. It was seen that in the other economic sectors greater use is made of technologies based on capital than on manpower. Nevertheless, in agribusiness the intense use of manpower is clear, especially if we analyze the share of the aggregates in total agribusiness employment (agriculture and livestock). These make intensive use of manpower in rural areas, with a share of 49.13%. However, although agribusiness is a major employer, the average income of this sector was lower than in the other sectors of the Brazilian economy. Besides, historically, we can analyze a significant decrease of rural population. In 2010, the Brazilian population was 190 million with a proportion of 15,6% of rural population. Forecasts made by the Brazilian Statistic Department (DIEESE) show that in 2050 the Brazilian population will be around 226 million with only 8% from rural areas. This estimated decrease is happening due to several factors, such as higher industrial concentration in urban areas (with increase of the demand for labor force), changes of the productive process in agriculture, fragility of supply of goods and services by the government in the rural areas (health, education, leisure, etc.), and increase of the technological level of rural activities [26].

In terms of foreign trade, the Brazilian economy is relatively closed, considering the amount of imports for the final demand of the industry and service sectors. In agribusiness, there is a more balanced relationship of imports between intermediate consumption and final demand. Regarding exports, in 2011, agribusiness accounted for 33.59% of the total GDP and was the only sector with a positive balance of trade, considering the amount of imports and exports.

The main contribution of this paper by assessing the economic dimension of agribusiness in the Brazilian economy is that it shows the strong link between agriculture and livestock, industry and services, and the other economic sectors. Thus, it can be said that the development of agribusiness is highly important to Brazilian economic structure and, therefore, to foster economic policies.

It is expected that the importance of agribusiness will remain in next years. According to long-term projections 2014/2015 to 2024/2025 published by Brazilian Ministry of Agriculture, Livestock and Food Supply, the grain production will increase at least 29% [27]. For meat production, an increase of at least 30.7% is expected. These growth rates of agricultural production should continue happening based on productivity. The Ministry's projections also highlight that exports and productivity gains should be the major growth factors over the next decade. There is a significant upward trend in the participation of Brazil in world trade of several commodities, such as soybeans, corn, beef, chicken, and pork. As noted, the Brazilian soybeans in 2024/25 are expected to have a share of world exports of 45.9% beef, 26.5% chicken, and meat 41.5%. Besides the importance in relation to these products, Brazil should maintain leadership in world trade in coffee and sugar.

For future research, we suggest analyzing and assessing the economic structure of the agribusiness of other Latin American countries.

## Figures and Tables

**Table 1:** Structure of input-output table.

Sectors/activities	Intermediate demand			Final demand	Total
	(1)	(2)	(3)		
Agriculture (1)	*x*_11_	*x*_12_	*x*_13_	*Y*_1_	*X*_1_
Industry (2)	*x*_21_	*x*_22_	*x*_23_	*Y*_2_	*X*_2_
Services (3)	*x*_31_	*x*_32_	*x*_33_	*Y*_3_	*X*_3_
Value added	*Z*_1_	*Z*_2_	*Z*_3_		
Total	*X*_1_	*X*_2_	*X*_3_		

Source: adapted from O'Connor and Henry [24] and Miller and Blair [25].

**Table 2:** Agribusiness in Brazilian GDP structure in basic and market prices-based, 2011.

Aggregates	Basic prices (without taxes)		
	Values^*∗*^	Aggregate share (%) in agribusiness GDP	Aggregate share (%) in Brazilian GDP
A: inputs	30,864.19	4.50	0.83
B: agriculture and livestock	190,570.00	27.78	5.12
C: agroindustry	185,262.59	27.01	4.98
D: distribution and services	279,249.88	40.71	7.51
*Agribusiness GDP (A + B + C + D) *	685,946.66	100.00	18.45
E: industry	713,864.76		19.20
F: industry services	95,859.51		2.58
G: services	2,223,173.07		59.78
*Remaining GDP of national economy (E + F + G) *	3,032,897.34		81.55
*Brazilian GDP (A + B + C + D + E + F + G)*	3,718,844.00		100.00

Aggregates	Market prices (with taxes)		
	Values^*∗*^	Aggregate share (%) in agribusiness GDP	Aggregate share (%) in Brazilian GDP

A: inputs	43,242.52	5.00	0.99
B: agriculture and livestock	199,177.00	23.03	4.55
C: agroindustry	308,580.93	35.68	7.05
D: distribution and services	313,768.51	36.28	7.17
*Agribusiness GDP (A + B + C + D) *	864,768.95	100.00	19.77
E: industry	1,058,982.65		24.21
F: industry services	127,487.88		2.91
G: services	2,323,525.52		53.11
*Remaining GDP of national economy (E + F + G)*	3,509,996.05		80.23
*Brazilian GDP (A + B + C + D + E + F + G)*	4,374,765.00		100.00

*Note*. ^*∗*^Values in millions of Brazilian Real (BR R$) currency.

**Table 3:** Indirect taxes and tax burden of Brazilian economy, 2011.

Aggregates	Values^*∗*^	Aggregate share (%)	Share (%) in the total	Tax burden^*∗∗*^
A:inputs	12,378.32	6.92	1.89	28.63
B: agriculture and livestock	8,607.00	4.81	1.31	4.32
C: agroindustry	123,318.34	68.96	18.80	39.96
D: distribution and services	34,518.63	19.30	5.26	11.00
*Agribusiness taxes (A + B + C + D) *	178,822.29	100.00	27.26	20.68
E: industry	345,117.89		52.62	32.59
F: industry services	31,628.37		4.82	24.81
G: services	100,352.45		15.30	4.32
*Remaining taxes of national economy (E + F + G) *	477,098.71		72.74	13.59
*Total indirect taxes (A + B + C + D + E + F + G)*	655,921.00		100.00	14.99

*Notes*. ^*∗*^Values in millions of Brazilian Real (BR R$) currency; ^*∗∗*^share (%) in the values of market prices (Table 2).

**Table 4:** Employment in Brazil and agribusiness, 2011.

Aggregates	Number of employees	Aggregate share (%)	Share (%) in the total
A: inputs	677,698	2.32	0.68
B: agriculture and livestock	14,378,446	49.13	14.44
C: agroindustry	6,617,198	22.61	6.65
D: distribution and services	7,590,534	25.94	7.62
*Agribusiness employment (A + B + C + D) *	29,263,877	100.00	29.39
E: industry	13,326,306		13.39
F: industry services	716,978		0.72
G: services	56,252,996		56.50
*Other employment in national economy (E + F + G) *	70,296,280		70.61
*Total employment (A + B + C + D + E + F + G)*	99,560,157		100.00

**Table 5:** Agribusiness in the Brazilian wage structure in terms of basic and market prices, 2011.

Aggregates	Basic prices			
	Values^*∗*^	Aggregate's share (%) in wage of agribusiness	Aggregate's share (%) in Brazilian wage	Aggregate's share (%) in GDP
A: inputs	11,532.26	4.91	0.79	37.36
B: agriculture and livestock	33,625.00	14.31	2.31	17.64
C: agroindustry	84,270.99	35.87	5.80	45.49
D: distribution and services	105,484.60	44.90	7.26	37.77
*Wage of agribusiness (A + B + C + D) *	234,912.85	100.00	16.16	34.25
E: industry	241,788.65		16.63	33.87
F: industry services	16,878.01		1.16	17.61
G: services	960,075.49		66.05	43.18
*Remaining wage of national economy (E + F + G) *	*1,218,742.15*		83.84	40.18
*Brazilian wage (A + B + C + D + E + F + G)*	*1,453,655.00*		100.00	39.09

Aggregates	Market prices			
	Average annual wage income per worker^*∗*^		Quantity of national minimum wages^*∗∗*^	

A: inputs	17,016.81		31.22	
B: agriculture and livestock	2,338.57		4.29	
C: agroindustry	12,735.15		23.37	
D: distribution and services	13,896.86		25.50	
*Wage of agribusiness (A + B + C + D) *	8,027.40		14.73	
E: industry	18,143.71		33.29	
F: industry services	23,540.48		43.19	
G: services	17,067.10		31.32	
*Remaining wage of national economy (E + F + G) *	*17,337.22*		31.81	
*Brazilian wage (A + B + C + D + E + F + G)*	*14,600.77*		26.79	

*Note*. ^*∗*^Values in millions of Brazilian Real (BR R$) currency.

^*∗∗*^Values in millions – national minimum wage of 2011 = BR R$ 545.

**Table 6:** Destination of Brazilian imports, 2011.

Destination	Agribusiness		Industry		Services		Total	
	Values^*∗*^	%	Values^*∗*^	%	Values^*∗*^	%	Values^*∗*^	%
Intermediate consumption	20,074	52.7%	140,650	64.6%	55,291	74.0%	216,015	65.4
Final demand	17,984	47.3%	77,097	35.4%	19,410	26.0%	114,491	34.6
Total	38,059	100.0%	217,747	100.0%	74,701	100.0%	330,507	100.0

*Note*. ^*∗*^Values in millions of US$.

**Table 7:** Brazilian balance of trade, 2011.

		Imports			Exports		Balance of trade
Sectors	Intermediate consumption	Final demand	Total				
	Values^*∗*^	Values^*∗*^	Values^*∗*^	%	Values^*∗*^	%	Values^*∗*^
Agribusiness	20,074	17,984	38,059	11.52	100,656	33.59	62,597
Industry	140,650	77,097	217,747	65.88	159,832	53.34	−57,916
Services	55,291	19,410	74,701	22.60	39,172	13.07	−35,529
Total	216,016	114,491	330,507	100.00	299,659	100.00	−30,848

*Note*. ^*∗*^Values in millions of US$.
